# Neural Mobilization for Lumbar Disc Prolapse: A Critical Appraisal

**DOI:** 10.1002/hsr2.71363

**Published:** 2025-10-09

**Authors:** Jie Hao

**Affiliations:** ^1^ Board‐Certified Geriatric Physical Therapy Specialist Southeast Colorado Hospital, Springfield Colorado USA

Dear Editor,

I read with great interest the recent article by Nahid et al. [1], “Effectiveness of Shacklock's Neural Mobilization for Acute and Sub‐Acute Lumbar Disc Prolapsed: A Randomized Controlled Trial.” While the topic is clinically important, several methodological and reporting issues merit clarification, as they may mislead readers regarding the effectiveness of the intervention.

First, the authors describe this as a “double‐blinded randomized controlled trial”, claiming that the assessor, physiotherapists, and participants were all blinded. However, this assertion is problematic and requires clarification. The intervention involved manual delivery of neural mobilization techniques, a hands‐on procedure that inherently reveals group allocation to both therapist and patient. The report further notes that only two of the four treating physiotherapists were trained in neural mobilization, which would make true blinding of therapists infeasible. Likewise, participant blinding is highly questionable, given the distinct nature of the interventions received. Therefore, blinding of therapists and participants should not be claimed without a detailed and credible explanation of how it was operationalized and verified.

Second, in Section 3.1 and Table 3, the authors report results from a Wilcoxon signed‐rank test and interpret these as between‐group differences. However, this test is designed for within‐group comparisons [2]. Despite this, the authors state that “most of the variables were significant differences found on the Posttest Dallas pain score between the two groups”, which is factually incorrect. No appropriate between‐group analysis is presented to support that claim. Figure 2, described as illustrating “posttest mean pain between both groups,” presents the same data as Table 3, which reflects pre‐ and posttreatment values likely from the experimental group alone. No data from the control group are displayed, and no between‐group comparisons are provided. Therefore, both the figure caption and the accompanying interpretation are misleading and may give readers the false impression that a between‐group analysis was performed and yielded significant differences.

Third, the authors report using the Dallas Pain Questionnaire (DPQ) as an outcome measure; however, the items presented in Table 3 do not correspond to the validated version of the DPQ. The original DPQ consists of 16 items organized into four domains: daily activities, work/leisure activities, emotional status, and social interest [3]. In contrast, the version used in this study focuses narrowly on position‐specific or task‐specific pain and omits the psychosocial dimensions. No rationale is provided for modifying the tool, nor is any evidence offered regarding the reliability, validity, or cross‐cultural adaptation of this version. This mislabeling not only limits the comparability of results to existing literature but also introduces the risk of measurement bias, as the unvalidated instrument may not accurately or consistently capture the intended constructs related to pain and function.

Fourth, the abstract and conclusion sections emphasize that Shacklock's neural mobilization had a “significant impact” on pain and disability. However, the study's own results show that no statistically significant differences were observed between groups post‐intervention on either the Dallas Pain Questionnaire or the Oswestry Disability Index. While within‐group improvements were noted in the experimental group, similar improvements were also seen in the control group, and between‐group comparisons did not support the added benefit of neural mobilization. Therefore, the addition of Shacklock's neural mobilization to standard physiotherapy did not result in superior outcomes, and its utility should not be interpreted as clinically effective based on this evidence. Overstating efficacy based on within‐group differences alone may mislead clinicians and readers.

In conclusion, while the exploration of neural mobilization in lumbar disc prolapse is timely and relevant, the interpretation of statistical findings, presentation of outcome measures, and overstatement of conclusions in this study warrant careful reconsideration. I encourage the authors to clarify these aspects to promote accurate reporting and evidence‐based clinical practice.

## Author Contributions

**Jie Hao:** conceptualization, methodology, writing – original draft, writing – review and editing.

## Conflicts of Interest

The author declares no conflicts of interest.

## Transparency Statement

The author confirms that the results and interpretations presented in this letter are transparent, and the methodology used in the study is clearly documented. All analyses were conducted without manipulation or selective reporting.

## Data Availability

The author has nothing to report.
